# ZacrosTools: A Python Library for Automated Preparation, Analysis, and Visualization of Kinetic Monte Carlo Simulations with Zacros

**DOI:** 10.1021/acs.jpca.5c02802

**Published:** 2025-07-14

**Authors:** Hector Prats

**Affiliations:** † Institute of Materials Chemistry, Technische Universität Wien, 1060 Vienna, Austria; ‡ Department of Chemistry, Physical & Theoretical Chemistry Laboratory, University of Oxford, South Parks Road, Oxford OX1 3QZ, U.K.

## Abstract

This paper presents ZacrosTools, a free and open-source Python library designed to simplify and automate the preparation and analysis of kinetic Monte Carlo (KMC) simulations with the widely used Zacros package. ZacrosTools provides a user-friendly and robust interface for building KMC models, automating the generation of Zacros input files, extracting and processing simulation data, and visualizing results through multiple plotting functionalities. The library benefits new users by simplifying model preparation and helping them avoid common mistakes while also being suitable to advanced users who wish to fine-tune complex KMC models and conduct comprehensive analyses. ZacrosTools is extensively documented with numerous examples available on ReadTheDocs and is publicly accessible on GitHub under the MIT license. Furthermore, it integrates continuous integration via GitHub Actions to facilitate seamless contributions from the user community.

## Graphical abstract

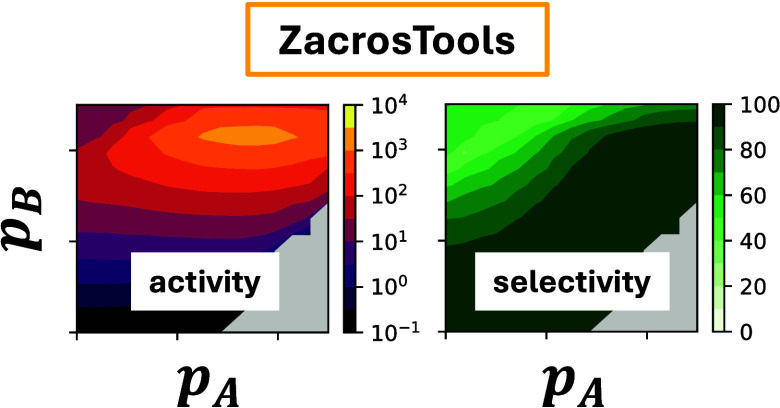

## 1 Introduction

Kinetic Monte Carlo (KMC) is a powerful stochastic simulation technique that finds applications across a wide range of fields, including surface science,^1^ materials growth,^2^ radiation damage in solids,^3^ battery degradation,^4^ and chemical kinetics.^5,6^ In particular, it has become the gold-standard method in multiscale modeling of reactive systems, where it bridges the gap between microscopic rate constants—often computed from Density Functional Theory (DFT) calculations—and macroscopic observables such as turnover frequencies (TOFs), product selectivity, and surface coverage. Analyzing the statistics of simulated events allows researchers to identify dominant reaction pathways and rate-determining steps as functions of reaction conditions (e.g., partial pressures and temperature). These insights are invaluable for both rational catalyst design and the deeper understanding of existing catalytic systems. Examples include catalytic processes on extended metal surfaces,^7–14^ supported clusters,^15–19^ and nanoparticles,^20,21^ diffusion processes of surface^22^ and subsurface species,^23^ oxide reduction processes,^24^ transformation of metal nanoalloys,^25^ and chemical etching,^26^ among others.

Among the various KMC packages available today, Zacros is the most widely used choice for modeling surface-catalyzed reactions.^27^ Written in modern Fortran, Zacros employs a graph-theoretical KMC formalism that handles many-body lateral interactions between adsorbed species with high computational efficiency.^28^ It incorporates coverage-dependent activation energies (e.g., via Bro̷nsted–Evans–Polanyi relations), dynamically updating reaction rate constants as local conditions change. Additionally, Zacros supports parallelization via OpenMP and MPI frameworks, enabling simulations on large spatial domains^29^ and being able to accommodate highly complex models with numerous possible adsorption sites, long-range interactions, and intricate reaction mechanisms on both periodic and non-periodic lattices. Furthermore, Zacros supports dynamic scaling of the rate constants of fast events to tackle the time-scale separation problem in KMC simulations. Given its versatility and robustness, Zacros has been widely adopted in both academic research and industrial applications.^7,10,14,15,17,18^

Despite its many strengths, preparing the Zacros input files and processing the output files can become cumbersome, particularly when conducting many simulations for a parameter scan (e.g., pressure and temperature sweeps) or when performing degree or rate control analysis. The manual process of editing text-based input files and extracting statistical data across multiple runs is time-consuming and error-prone. ZacrosTools is designed to automate these tasks, simplifying the model construction, input file generation, data analysis and visualization of results. The main goal of ZacrosTools is to make Zacros more accessible to researchers and to offer a common framework to the Zacros user community for developing and sharing new analysis tools. To date, ZacrosTools has already been employed in at least one published study,^15^ where its utility for streamlining large KMC parameter scans is demonstrated.

Zacros users might already be familiar with Zacros-Post, a graphical user interface (GUI) for visualizing the results of individual simulations.^30^ While Zacros-Post is particularly useful for users who prefer an interactive, GUI-based environment to quickly inspect outputs such as species coverages, reaction rates, or event process statistics from a single simulation, ZacrosTools offers a complementary, script-based approach focused on large-scale analysis that can be integrated into automated workflows, making it particularly suited for high-throughput applications, such as sensitivity analyses or parameter sweeps (e.g., pressure or temperature).

In this paper, we present the main features of ZacrosTools, illustrating how it facilitates the construction of KMC models, generation of Zacros input files, and extraction and visualization of simulation results. Code snippets are provided to demonstrate each step of a typical workflow. As examples, we consider CO oxidation, the water–gas shift (WGS) reaction, and the dry reforming of methane (DRM). These are prototypical and industrially relevant reactions in heterogeneous catalysis, widely studied due to their importance in pollution control and syngas production.^31^ All three have been extensively modeled using KMC in the literature and serve as representative cases to illustrate the capabilities of ZacrosTools.

## 2 Methods

ZacrosTools is an object-oriented Python library distributed under the MIT license, which guarantees its users to freely use, share, and modify the software. The current stable version of ZacrosTools is 2.4, which is publicly available on PyPi,^32^ while the most recent development version can be accessed on GitHub.^33^ Extensive documentation, including beginner tutorials and practical examples, is hosted on ReadTheDocs.^34^ ZacrosTools relies on four core Python libraries: numpy and pandas for data analysis, scipy for physical constants, and matplotlib for plotting. This library is actively developed with ongoing enhancements driven by community feedback and is continuously tested and built via GitHub Actions, including automated test-coverage reporting through Codecov.^35^ Readers are encouraged to consult the GitHub repository to explore the latest features, updates, and documentation as the tool continues to evolve.

## 3 Results and Discussion

### 3.1 Preparation of Input Files

Zacros simulations require several input files that define the lattice, adsorbed and gas-phase species, reaction mechanisms, and global simulation parameters, such as temperature, partial pressures, and simulation length. ZacrosTools simplifies creating these inputs by breaking the model into four main components: (i) a *lattice model* that defines site arrangements and connectivity, (ii) a *gas model* containing thermochemical properties of each gas-phase species, (iii) an *energetics model* specifying energies of adsorbed clusters used in the cluster expansion, and (iv) a *reaction model* that includes all elementary steps, activation barriers, and vibrational energies. The following sections explain how each of these components is built, and how they are combined into a full KMC model which can then automatically generate all Zacros input files.

#### 3.1.1 Lattice Model

The first step in building a KMC model is to define the lattice model, represented in ZacrosTools as an instance of the LatticeModel class. For periodic systems, users can use a variety of arguments to specify a unit cell, its site types, and the neighboring structure, which determines site connectivity. The neighboring structure can either be explicitly defined or automatically generated from a distance cutoff dictionary. Figure 1A demonstrates how ZacrosTools can create the lattice model for HfC(001) used in ref 15 by automatically determining neighboring structures.

**Figure 1:**
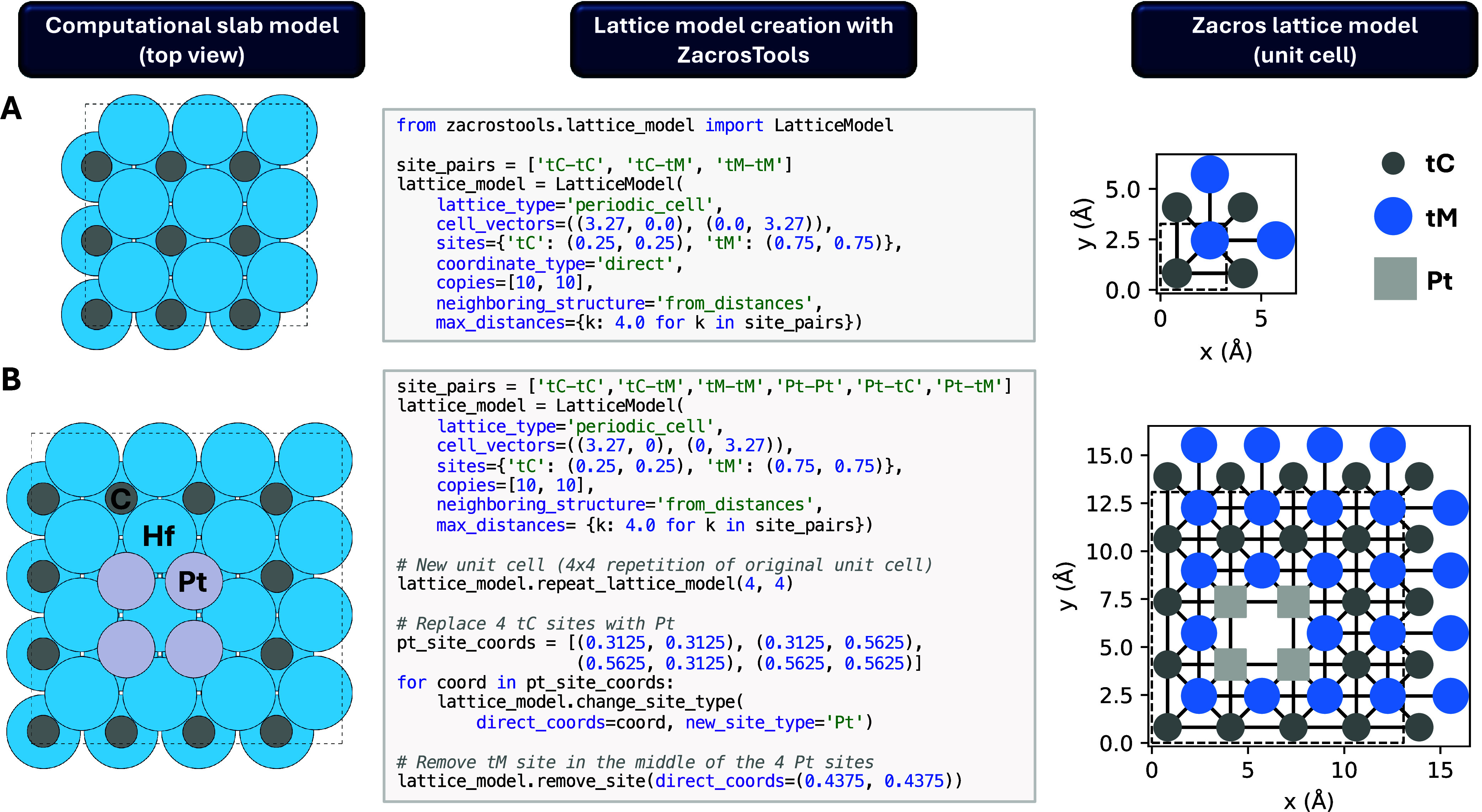
Creation of two custom periodic lattice models for (A) a HfC(001) slab and (B) a Pt_4_ cluster supported on a HfC(001) slab using ZacrosTools.

To facilitate the creation of more complex lattice structures, ZacrosTools includes methods to modify a base lattice, such as repeating the unit cell, removing specific sites, or changing site types. These capabilities allow users to construct complex lattice models in a simple way. Figure 1B illustrates how to create a lattice model for the Pt_4_ cluster supported on HfC(001) used in ref 15. To verify that the lattice model has been created as intended, ZacrosTools provides a plot_custom_lattice function, which produces a graphical representation of the lattice_input.dat file. For simpler cases, ZacrosTools also supports the creation of default Zacros periodic lattices (triangular, rectangular, or hexagonal) by specifying the desired lattice type. Further details on lattice model creation can be found in Section S1 of the Supporting Information (SI).

#### 3.1.2 Gas, Energetics, and Reaction Models

The remaining pieces required to build a MC model include the gas, energetics, and reaction models. These models are created as an instance of the GasModel, EnergeticsModel, and ReactionModel classes, respectively. In the gas model, each species is defined with properties such as molecular weight, symmetry number, and formation energy. Additional details, such as ground-state degeneracy, can be optionally specified. The energetics model defines cluster formation energies and site configurations, incorporating factors like lateral interactions and graph multiplicities to account for symmetry effects. The reaction model describes elementary steps using initial and final configurations, activation energies, vibrational energies, and connectivity constraints. ZacrosTools automatically computes pre-exponential factors based on transition state theory (TST), adjusting for different reaction types such as surface reactions, Eley–Rideal mechanisms, and activated adsorption.

The simplest way to create these models is by initializing them from a Python dictionary using the from_dict method. Alternatively, ZacrosTools allows users to create these models from Pandas DataFrames or CSV files using the from_df and from_csv methods. Further details and examples on creating gas, energetics and reaction models can be found in Section S2 of the Supporting Information. Finally, the TST-derived expressions used in ZacrosTools to calculate the pre-exponential factors are summarized in Section S3 of the Supporting Information.

#### 3.1.3 Putting All Together: The KMC Model

After defining the lattice, gas, energetics, and reaction models, they are combined into a KMC model, represented as an instance of the KMCModel class. Once the KMCModel is created, Zacros input files can be generated using the create_job_dir method, which requires parameters such as temperature, gas-phase pressures, reporting schemes, and stopping criteria. The code snippet in Scheme 1 below shows how to assemble a simple model for CO adsorption, desorption, and diffusion on a hexagonal periodic lattice, and how to write the Zacros input files for a simple temperature and pressure scan. In this example, a 10 × 10 hexagonal periodic lattice is used, the gas model contains a single CO species, the energetics model accounts for single-body formation energy and lateral interactions between neighboring CO molecules, and the reaction model defines the adsorption and diffusion steps. Additionally, ZacrosTools provides options for manual and automatic rate constant scaling, which help address time-scale separation issues in KMC simulations. Users can manually scale rate constants for specific steps or enable automatic scaling algorithms. Further information on all the arguments of the create_job_dir method, including how to enable and control automatic scaling of rate constants, is provided in Section S4 of the Supporting Information.

**Scheme 1:**
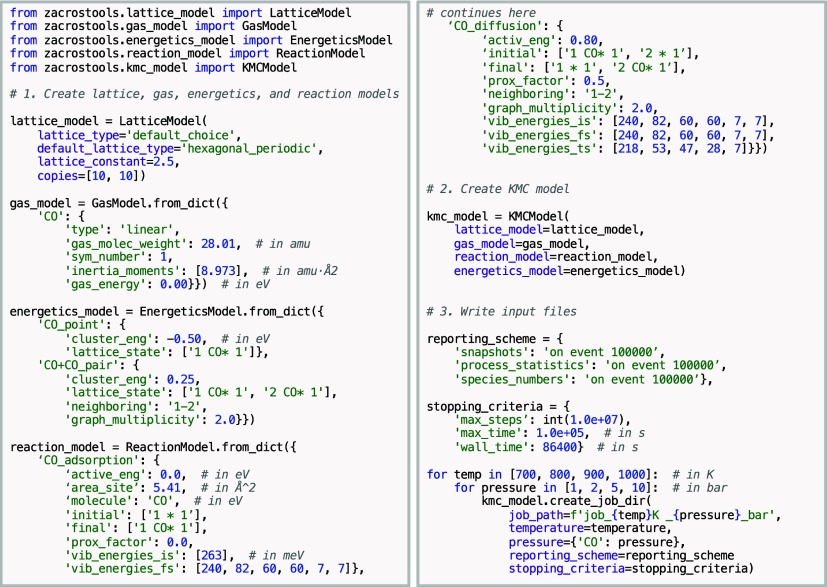
Building a KMC model for CO adsorption/desorption and diffusion on a hhexagonal periodic lattice, and generating Zacros input files for a temperature and pressure scan

### 3.2 Parsing Output Files

#### 3.2.1 Extracting Data from a Single Simulation

The KMCOutput class provides a convenient interface to load simulation data from output files, compute averages, and extract macroscopic observables such as coverages, TOFs and selectivities. This functionality also works with unfinished simulations. A KMCOutput instance can be created by specifying the folder containing the Zacros output files (e.g., general_output.txt, specnum_output.txt, etc.). Additionally, one can define an analysis range (in terms of simulation time or number of KMC events) and a weighting scheme. The main KMCOutput arguments are job_path (directory containing the Zacros output files), analysis_range (a 2-element list specifying the portion of the simulation to consider for analysis, in %), range_type (can be “time” for simulation time or “nevents” for number of KMC events), and weights (weighting scheme used for averaging, possible values are “time”, “nevents”, or “None”). Once a KMCOutput instance is created, its attributes and methods can be used to extract data, as listed in Table S2 in the SI. The code snippet in Scheme 2 demonstrates typical usage, including printing the TOF of each gas-phase species, computing the selectivity of CH_4_ with respect to CO_2_ and CH_3_OH, and reporting the average coverage for all surface-adsorbed species.

**Scheme 2:**
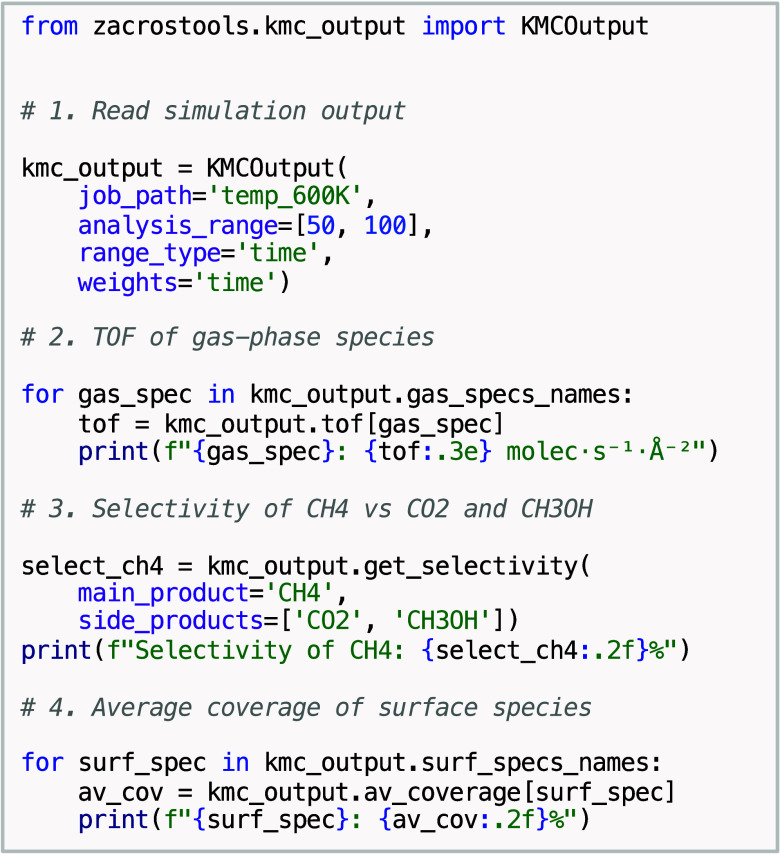
Extracting simulation data from the output files

#### 3.2.2 Extracting Data from a Large Set of Simulations

When running numerous simulations, manually extracting data from each directory can be tedious and prone to error. ZacrosTools addresses this challenge with the read_scan function, which reads results from all simulations in a specified directory and returns a Pandas DataFrame containing the parsed output from each run. This function accepts the same arguments as KMCOutput, including analysis_range, range_type, and weights, and uses scan_path instead of job_path. An example is provided in Section S5 of the Supporting Information.

#### 3.2.3 Steady State Analysis and Automatic Detection of Issues

KMC simulations are typically run until the system has reached steady state and collected enough statistics during the production phase to ensure the measured observables have reached stable mean values. As shown in Section 3.2.1, one can then compute macroscopic observables such as TOF and coverage from the production phase only selecting the appropriate analysis_range in the KMCOutput class. However, in large parameter scans, it becomes impractical to manually select the appropriate analysis range for each simulation. A simple workaround is to choose a uniform cutoff for all simulations (e.g., the last 50% of simulated time), but this may not be suitable for runs with prolonged equilibration phases. To address this issue, ZacrosTools provides the detect_issues function, which automatically determines whether the chosen analysis_range is appropriate by examining trends in the lattice energy and the linearity of simulation time with respect to the number of events. Details on this function are given in Section S6 of the Supporting Information.

### 3.3 Plotting Results

This section shows how to visualize KMC simulation results in ZacrosTools. Section 3.3.1 focuses on plotting output from a single simulation (e.g., surface coverage and gas-phase molecules produced), while Section 3.3.2 demonstrates how to generate 2D heatmaps for large parameter scans.

#### 3.3.1 Plotting Single Simulation Results

A logical first step in analyzing a catalytic system is to inspect how coverages and production evolve over a single KMC simulation. In ZacrosTools, users can employ the data extracted via KMCOutput (see Section 3.2.1) and then plot it using matplotlib. Besides coverage and gas production, other quantities like event frequencies or stiffness coefficients (if stiffness scaling of rate constants has been used) can also be visualized.

The simplest way to plot simulation results is to extract the simulation data from the KMCOutput object and then plot the desired data using matplotlib. Moreover, ZacrosTools includes a dedicated plot_event_frequency function for plotting the event frequencies of the different elementary steps in the reaction model. Figure 2 shows how to generate plots for coverage, gas-phase production, and event frequencies in the water–gas shift (WGS) KMC model included in the *examples* folder of Zacros. Although simple, the model includes two possible reaction pathways, and analyzing the event frequencies is necessary to determine the dominant one.

**Figure 2:**
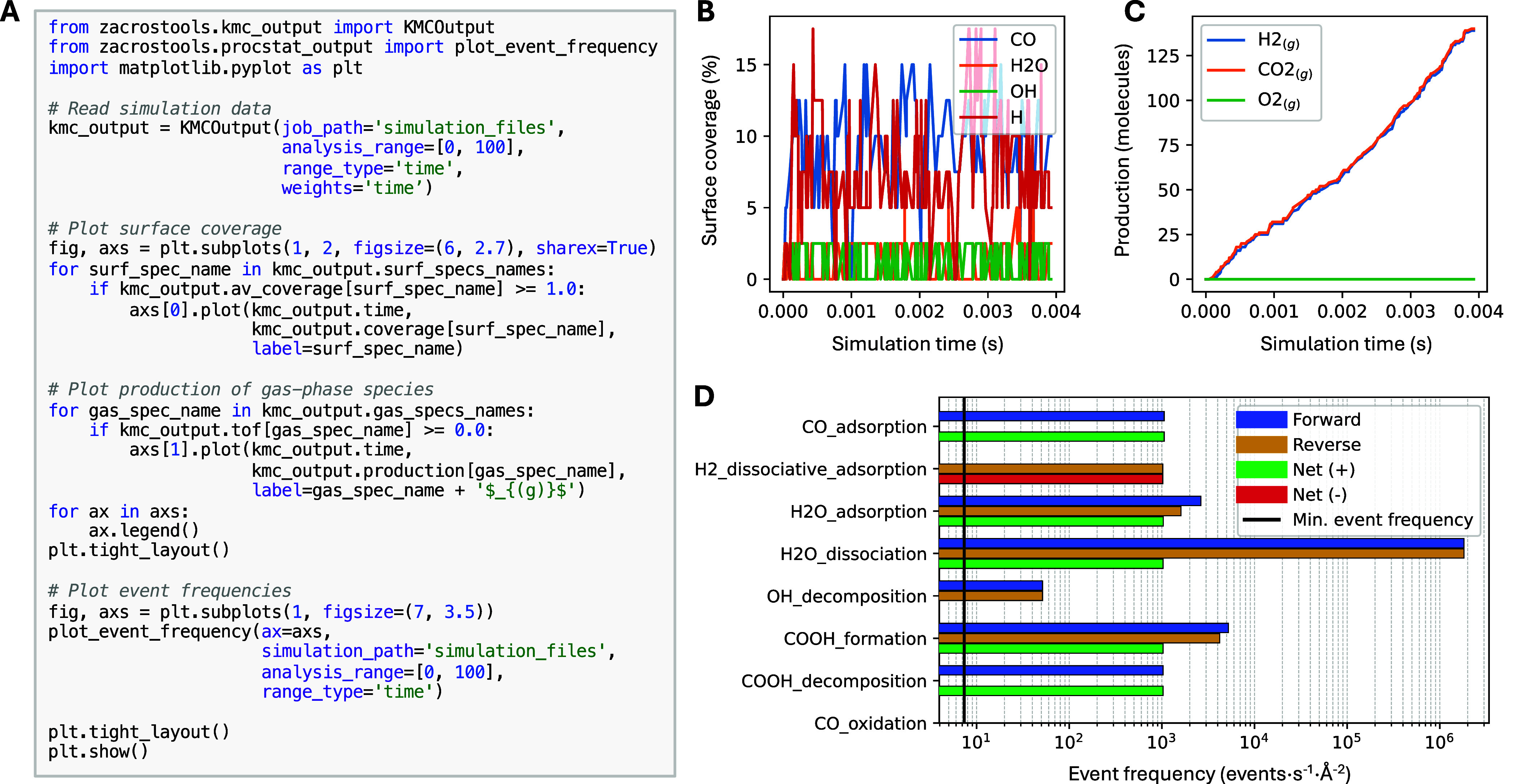
(A) Representative code snippet for plotting results from a single simulation, (B) coverage of surface species over time, (C) production of gas-phase species over time, and (D) event frequencies of the different reaction channels.

#### 3.3.2 Plotting Heatmaps

When multiple KMC simulations are run at varying pressures or temperatures, two-dimensional heatmaps offer a concise overview of the main results (e.g., turnover frequency, coverage, selectivity) across parameter space. ZacrosTools includes several specialized functions for creating heatmaps, such as plot_tof, plot_selectivity, plot_phasediagram, and so on, which differ in the magnitude plotted in the *z* variable. For instance, plot_tof generates a 2D heatmap of the TOF, while plot_phasediagram produces a phase diagram showing what is the dominant surface species as a function of the reaction conditions.

These functions all require specifying which parameters appear on the *x*- and *y*-axes (e.g., total pressure, partial pressures of specific gases, or temperature). Figure 3 presents an example of heatmap plots that can be generated with ZacrosTools, including TOF maps for multiple products, coverage maps for various site types, and phase diagrams and selectivity maps derived from the same data. The Python code used to create these heatmaps is provided in Section S7 of the Supporting Information.

**Figure 3:**
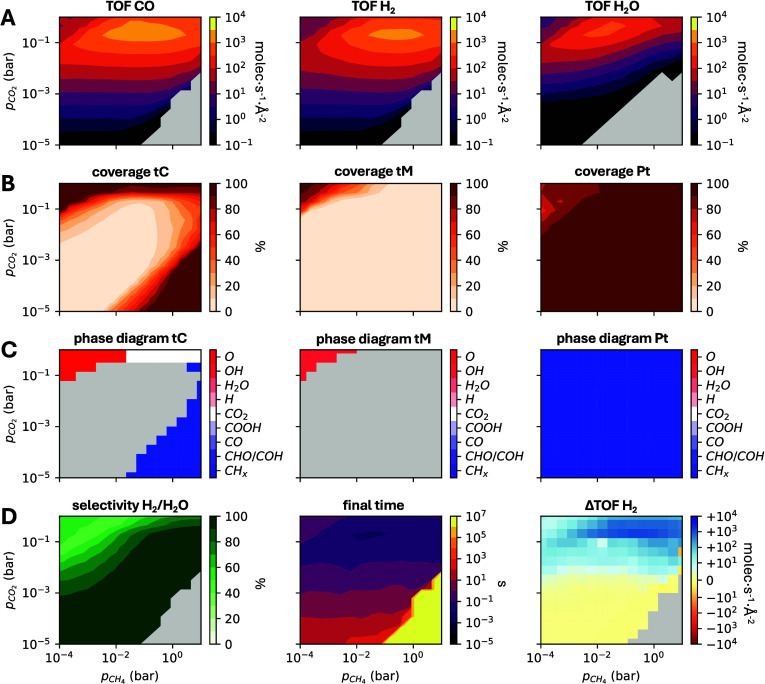
Example heatmap plots created with ZacrosTools showing the (A) TOF for three selected gas-phase species, (B) total coverage of surface species on three selected surface site types, (C) kinetic phase diagrams on three selected surface site types, and (D) selectivity for a specific product (left), the final simulation time (middle) and the change in TOF between two scans for a specific product (right). The simulation results are taken from ref 15 and correspond to a KMC model of the dry reforming of methane on Pt/HfC.

## 4 Conclusions

ZacrosTools provides a robust and user-friendly interface for the Zacros KMC package, speeding up the preparation of KMC simulations, minimizing the potential for input errors, and offering multiple classes and functions to parse and analyze output files and visualize the results. Among other things, this open-source Python library provides tools for building complex lattice models, detecting potential issues in finished simulations, parsing and storing the results of large KMC parameter scans into a CSV file, and generating 2D heatmap plots of the TOF, selectivity or coverage as a function of chosen operating conditions. Furthermore, ZacrosTools also invites the Zacros user community to collaborate thanks to continuous integration via GitHub Actions, active development, and automated test-coverage reporting through Codecov. Users can easily contribute new functionalities, enhance existing features, and collectively ensure that ZacrosTools continues to evolve as a valuable resource for advancing research in heterogeneous catalysis and surface chemistry.

## Supplementary Material



## Data Availability

The source code of ZacrosTools is freely available under the open-source MIT license on GitHub at https://github.com/hprats/ZacrosTools. The package is also published on the Python Package Index (PyPI) at https://pypi.org/project/zacrostools/ and can be installed via PIP using this command: pip install zacrostools. The documentation is hosted at https://zacrostools.readthedocs.io/en/latest/.

## Notes

Additional details on lattice, gas, energetics, and reaction models (Sections S1 and S2); calculation of pre-exponential factors (Section S3); additional details and examples on writing input files and extracting output data (Sections S4 and S5); steady state analysis and automatic detection of issues (Section S6); and additional details and examples on plotting 2D heatmaps (Section S7) (PDF)
