# Supplementary material for: ZacrosTools: A Python Library for Automated Preparation, Analysis, and Visualization of Kinetic Monte Carlo Simulations with Zacros
Source: J Phys Chem A. 2025 Jul 14;129(29):6608–14. doi: 10.1021/acs.jpca.5c02802 (PMC12302213; doi:10.1021/acs.jpca.5c02802)
Supplement: Supplementary file 1 [file jp5c02802_si_001.pdf]

ZacrosTools: A Python Library for Automated Preparation, Analysis, and Visualization of Kinetic Monte Carlo Simulations with Zacros

Hector Prats*[a,b]

[a] *Institute of Materials Chemistry, Technische Universität Wien, 1060 Vienna, Austria*

[b] *Department of Chemistry, Physical & Theoretical Chemistry Laboratory, University of Oxford, South Parks Road, Oxford OX1 3QZ, U.K*

*Corresponding author: Hector Prats (hector.prats@tuwien.ac.at)

Section S1: Additional details on lattice models. **S2**

Section S2. Additional details on gas, energetics and reaction models **S3**

Section S3. Calculation of pre-exponential factors **S5**

Section S4. Writing input files **S7**

Section S5. Extracting output data **S8**

Section S6. Steady state analysis and automatic detection of issues **S10**

Section S7. Plotting heatmaps **S12**

REFERENCES **S14**

Section S1. Additional details on lattice models

S1.1 Arguments

A periodic lattice can be created setting `lattice_type='periodic_cell'` and providing the following arguments: `cell_vectors` (a tuple of unit vectors defining the unit cell), `sites` (a dictionary mapping site types to their coordinates), `coordinate_type` (either `'direct'` or `'cartesian'`), `copies` (the number of copies of the unit cell in horizontal and vertical directions), and `neighboring_structure` (dictionary mapping site-pair labels to a list of Zacros relationship keywords such as `self`, `north`, or `east`). Alternatively, ZacrosTools can automatically generate the neighboring structure by setting `neighboring_structure='from_distances'` and providing a distance cutoff dictionary in Å, along with the additional argument `max_distances`.

To facilitate the creation of more complex lattice models, ZacrosTools includes the methods `repeat_lattice_model(m, n)` (repeats the unit cell m and n times in the horizontal and vertical directions), `remove_site()` (removes specified sites), and `change_site_type()` (reassigns a site's type). An example is shown in Figure 1B.

ZacrosTools can also generate a `lattice_input.dat` file for a default periodic lattice by setting `lattice_type='default_choice'` and specifying three additional parameters. First, a `default_lattice_type` must be chosen from among `'triangular_periodic'`, `'rectangular_periodic'`, or `'hexagonal_periodic'`. Next, a `lattice_constant` must be defined (in Å), and finally the `copies` argument must be provided to determine how many times the cell is repeated in each direction. Code snippet S1 shows how to create a 10×10 default hexagonal periodic lattice:

```
from zacrostools.lattice_model import LatticeModel

lattice_model = LatticeModel(
    lattice_type='default_choice',
    default_lattice_type='hexagonal_periodic',
    lattice_constant=2.5,
    copies=[10, 10])
```

Code snippet S1. Creating a 10×10 default hexagonal periodic lattice

Additional information on creating lattice models with ZacrosTools can be found in the documentation.[1]

S1.2 Plotting a periodic lattice

The `lattice_input.dat` file of a periodic lattice can be visualized using the `plot_periodic_lattice()` function. This function requires the `filename` argument (the path to the lattice input file) and `site_styles`, a dictionary mapping site type names to style dictionaries. Each style dictionary may include `'color'`, `'marker'`

and `'size'`. In addition, one can also specify the optional arguments `ax` (matplotlib axes object on which to plot the lattice; if not provided, a new figure and axes are created) and `line_width` (width of the lines used to plot the connectivity's between sites and the unit cell boundaries). The code snippet below shows how to plot the lattice model for the HfC(001) slab shown in Figure 1A:

```
import matplotlib.pyplot as plt
from zacrostools.plot_periodic_lattice import plot_periodic_lattice

site_styles = {
    'tM': {'color': 'dodgerblue', 'marker': 'o', 'size': 250},
    'tC': {'color': 'grey', 'marker': 'o', 'size': 150}}

fig, ax = plt.subplots(1, figsize=(2.0, 2.0))
plot_periodic_lattice(
    filename='lattice_input.dat',
    ax=ax,
    site_styles=site_styles,
    line_width=1.5)
plt.tight_layout()
plt.show()
```

Code snippet S2. Plotting the lattice model for HfC(001) as shown in Figure 1A.

Section S2. Additional details on gas, energetics and reaction models

S2.1 Gas model

Each key in the gas model dictionary corresponds to a gas-phase species name, and its associated value is another dictionary of molecule properties. Mandatory keys include the molecule `type`, which can be set to `'linear'` or `'non_linear'`, `gas_molec_weight` in atomic mass units (amu), `sym_number` (the molecule's symmetry number), `inertia_moments` in units of amu·Å², and `gas_energy` (formation energy in eV). An optional `degeneracy` key can be included to specify the ground state degeneracy of the molecule, defaulting to 1 if not provided.

S2.2 Energetics model

In the energetics model, each key identifies a cluster, and its value is another dictionary of cluster properties. Mandatory keys are `cluster_eng` (the formation energy in eV), and `lattice_state` (a list of strings representing the cluster configuration in Zacros format). Optional keys include `site_types` (to define the type of each site, which is necessary if a custom periodic lattice is used), `neighboring` (the connectivity between

sites, defaulting to `None` if not provided), `angles` (angle constraints in Zacros format), and `graph_multiplicity` (the cluster's symmetry number, defaulting to `None`).

S2.3 Reaction model

Each key in the reaction model identifies a unique elementary reaction step. Its value is another dictionary of step properties, where the required keys include `initial` and `final` (lists of strings representing the initial and final configurations in Zacros format), `activ_eng` (activation energy in eV), `vib_energies_is` (a list of vibrational energies for the initial state in meV), and `vib_energies_fs` (a list of vibrational energies for the final state in meV). Additional required keys depend on the type of reaction step. Adsorption steps must include `molecule` (the gas-phase molecule involved) and `area_site` (the adsorption site area in Å^2), whereas activated steps (such as activated adsorption or surface reactions) also require `vib_energies_ts` (a list of vibrational energies for the transition state in meV). Optional keys such as `site_types` (if a custom periodic lattice is used), `neighboring` (connectivity between sites), `prox_factor` (proximity factor), `angles` (angle constraints), and `graph_multiplicity` (the step's symmetry factor) can be included to refine the reaction model.

S2.4 Creating models using different input formats

As mentioned in Section 3.1 of the main text, the gas, energetics and reaction models can also be created from a Pandas dataframe or from a `.csv` file using `from_df()`or `from_csv()` methods. In this case, the row indices correspond to the names of the gas-phase species, cluster or elementary steps, respectively, and the column names correspond to the keys discussed above for the creation from a dictionary. The following code shows three equivalent ways of creating the gas model for the example in Code snippet 1:

```
import pandas as pd
from zacrostools.gas_model import GasModel

# Option 1: from Python dictionary
gas_data = {
    'CO': {
        'type': 'linear',
        'sym_number': 1,
        'inertia_moments': [8.973],  # amu·Å2
        'gas_energy': 0.00}}  # eV
gas_model = GasModel.from_dict(gas_data)

# Option 2: from Pandas dataframe
gas_data = {
    'type': ['linear'],
    'sym_number': [1],
    'inertia_moments': [[8.973]],
    'gas_energy': [0.00]}
df = pd.DataFrame(gas_data, index=['CO'])
gas_model = GasModel.from_df(df)

# Option 3: from a .csv file (Table S1)
gas_model = GasModel.from_csv('gas_data.csv')
```

Code snippet S3. Three different ways of creating a gas model with ZacrosTools.

Table S1 shows the contents of the `.csv` file required to create the same gas model as in the previous example.

Table S1. Contents of the `.csv` file required to create a gas model for a CO gas-phase species.

	type	sym_number	inertia_moments	gas_energy
CO	linear	1	[8.973]	0.00

Section S3. Calculation of pre-exponential factors

ZacrosTools uses standard TST-derived expressions to calculate the pre-exponential factors from first-principles data. The partition functions are calculated assuming the validity of the ideal gas model for gas-phase species and the harmonic oscillator model for surface species. Additional models such as the hindered-translator/hindered-rotor (HT/HR) model[2] will be implemented in future releases.

In Zacros, the configuration-dependent rate constants for the elementary steps are calculated at each KMC step from Arrhenius relationships:

$$k_{fwd}(\sigma) = A_{fwd} \cdot exp\left(-\frac{E^{\ddagger}_{fwd}(\sigma)}{k_B T}\right) \quad (1)$$

$$k_{rev}(\sigma) = A_{rev} \cdot exp\left(-\frac{E^{\ddagger}_{rev}(\sigma)}{k_B T}\right) \quad (2)$$

where $k_{fwd}(\sigma)$ and $k_{rev}(\sigma)$ are the forward and reverse rate constants, respectively, for the lattice configuration σ, $E^{\ddagger}_{fwd}(\sigma)$ and $E^{\ddagger}_{rev}(\sigma)$ are the forward and reverse activation energies, respectively, for the lattice configuration σ, and A_{fwd} and A_{rev} are the forward and reverse pre-exponential factors, calculated at zero-coverage limit and with the adsorbed species at infinite separation. The configuration-dependent activation energies in Eqs. 1-2 are parameterized in terms of a Brønsted-Evans-Polanyi (BEP) relationship using the calculated activation energies at zero-coverage limit (i.e., specified using the `active_eng` keyword in the ZacrosTools reaction model) and the cluster expansion Hamiltonian. The reader is referred to the Zacros manual for further details on the BEP parameterization.

The pre-exponential factors are assumed to be independent of the configuration, and ZacrosTools uses different expressions depending on the nature of the elementary step. For a surface reaction of the type X+Y↔Z,

the partition functions q have only vibrational components (i.e., harmonic oscillator model) and the pre-exponential factor is calculated as:

$$A_{surf,fwd} = \frac{k_B T}{h} \frac{q_{vib}^{\ddagger}}{q_{vib,X} \cdot q_{vib,Y}} \tag{3}$$

$$A_{surf,rev} = \frac{k_B T}{h} \frac{q_{vib}^{\ddagger}}{q_{vib,Z}} \tag{4}$$

The vibrational partition functions for the different species and the transition state are calculated as a product of contributions from each vibrational mode:

$$q_{vib,i} = \prod_k \frac{exp\left(-\frac{h \cdot v_k}{2k_B T}\right)}{1 - exp\left(-\frac{h \cdot v_k}{k_B T}\right)} \tag{5}$$

where v_k is the vibrational energy of vibrational mode k. In ZacrosTools, these vibrational energies are specified in meV as lists using the keywords `'vib_energies_is'`, `'vib_energies_fs'` and `'vib_energies_ts'` in the reaction model for the initial, final and transition states, respectively.

For an Eley-Rideal reactions of the type $X_{(g)}$+Y↔Z, the partition function for the gas-phase species have not only vibrational components but also translational, rotational and electronic. In this case, the following expression can be derived:[3]

$$A_{ER,fwd} = \frac{p_X A}{\sqrt{2\pi \cdot m_X \cdot k_B T}} \frac{q_{vib}^{\ddagger}}{q_{trans2D,X_{(g)}} \cdot q_{rot,X_{(g)}} \cdot q_{vib,X_{(g)}} \cdot q_{el,X_{(g)}} \cdot q_{vib,Y}} \tag{6}$$

$$A_{ER,rev} = \frac{k_B T}{h} \frac{q_{vib}^{\ddagger}}{q_{vib,Z}} \tag{7}$$

where p_X and m_X are the partial pressure and molecular mass of $X_{(g)}$, A is the effective area of the active site, $q_{trans2D,X_{(g)}}$ is the 2D translational partition function over a unit cell of the surface, $q_{rot,X_{(g)}}$ is the rotational partition function, and $q_{el,X_{(g)}}$ is the electronic partition function:

$$q_{trans2D,X_{(g)}} = A \frac{2\pi \cdot m_X \cdot k_B T}{h^2} \tag{8}$$

$$q_{rot,X_{(g)}} = \begin{cases} \frac{8\pi^2 \cdot I \cdot k_B T}{\sigma \cdot h^2} & \text{(linear molecules)} \\ \frac{\sqrt{\pi I_a I_b I_c}}{\sigma}\left(\frac{8\pi^2 \cdot k_B T}{h^2}\right)^{3/2} & \text{(nonlinear molecules)} \end{cases} \tag{9}$$

$$q_{el,X_{(g)}} \approx g_0 \tag{10}$$

where I is the moment of inertia, σ is the symmetry number, and g_0 is the degeneracy of the ground electronic state.

Finally, for activated and non-activated adsorption steps of the type $X_{(g)} \leftrightarrow X$, the pre-exponential factors are calculated as follows:

$$A_{ads(act),fwd} = \frac{p_X A}{\sqrt{2\pi \cdot m_X \cdot k_B T}} \frac{q_{vib}^{\ddagger}}{q_{trans2D,X_{(g)}} \cdot q_{rot,X_{(g)}} \cdot q_{vib,X_{(g)}} \cdot q_{el,X_{(g)}}} \quad (11)$$

$$A_{ads(act),rev} = \frac{k_B T}{h} \frac{q_{vib}^{\ddagger}}{q_{vib,X}} \quad (12)$$

$$A_{ads(non-act),fwd} = \frac{p_X A}{\sqrt{2\pi \cdot m_X \cdot k_B T}} \quad (13)$$

$$A_{ads(non-act),rev} = \frac{k_B T}{h} \frac{q_{trans2D,X_{(g)}} \cdot q_{rot,X_{(g)}} \cdot q_{vib,X_{(g)}} \cdot q_{el,X_{(g)}}}{q_{vib,X}} \quad (14)$$

Section S4. Writing input files

After creating a `KMCModel` instance, one can automatically generate the four main Zacros input files — `simulation_input.dat`, `lattice_input.dat`, `energetics_input.dat`, and `mechanism_input.dat`— for specified conditions using the `create_job_dir()` method. The following parameters are required: `job_path` (directory where the input files will be written), `temperature` (in K), `pressure` (dictionary of partial pressures of gas-phase species, in bar), `reporting_scheme` (dictionary containing the values for the Zacros keywords `snapshots`, `process_statistics` and `species_numbers`, which control how often the information is written in the output files), and `stopping_criteria` (dictionary containing the values for the Zacros keywords `max_steps`, `max_time` and `wall_time`, which determine the conditions to end the simulation). Optionally, users can select a specific random seed by using the `random_seed` attribute and control the number of significant figures used for energies, pre-exponential factors, and coordinates by means of the `sig_figs_energies`, `sig_figs_pe`, and `sig_figs_lattice` attributes, respectively.

The `create_job_dir()` method also contains optional attributes used to enable and control the manual or automatic rate constant scaling to tackle the time-scale separation between elementary steps, which is commonly needed in KMC simulations. Manual scaling of the rate constants of specific elementary steps can

be done by specifying the `manual_scaling` attribute, which is a dictionary of step names as keys and their corresponding scaling factors as values, e.g. `{'CO_diffusion': 1.0e-02}`. Alternatively, or in addition to manual scaling, the automatic stiffness scaling algorithms implemented in Zacros can be enabled and controlled with ZacrosTools using the following attributes: `stiffness_scaling_algorithm` (algorithm used for stiffness scaling; in the current Zacros 4.0 version, only `'legacy'` is allowed, but more algorithms will become available in further releases), `stiffness_scalable_step` (steps that will be marked as stiffness scalable, can be provided as a list of names or the string `'all'` to indicate that all steps are stiffness scalable), and `stiffness_scaling_tags` (dictionary of keywords controlling the stiffness scaling algorithm and their corresponding values). Code snippet S4 provides an example of enabling stiffness scaling of rate constants. More information on this topic is provided in the official documentation.[1]

```
# Create the KMC model here

kmc_model.create_job_dir(
    job_path='job_800K_1.0_bar',
    temperature=800,
    pressure={'CO': 1.0},
    reporting_scheme={
        'snapshots': 'on event 100000',
        'process_statistics': 'on event 100000',
        'species_numbers': 'on event 100000'},
    stopping_criteria={
        'max_steps': 1.0e+07,
        'max_time': 1.0e+05,
        'wall_time': 86400},
    stiffness_scaling_algorithm='legacy',
    stiffness_scalable_steps='all',
    stiffness_scaling_tags={
        'check_every': 1000,
        'min_separation': 100.0,
        'max_separation': 200.0,
        'tol_part_equil_ratio': 0.05,
        'stiffn_coeff_threshold': 1.0,
        'scaling_factor': 5.0},
)
```

Code snippet S4. Using the stiffness scaling algorithm.

Section S5. Extracting output data

Simulation data can be obtained from an instance of `KMCOutput`, as described in Section 3.2.1. Table S2 summarizes the attributes and methods available for extracting relevant information, such as coverage, energy, final production counts, turnover frequencies, and more. For multiple simulations, the `read_scan()` function is often more efficient. This function reads data from all simulations in a specified directory and compiles the

results into a single Pandas DataFrame, facilitating further analysis or export to a CSV file. Code snippet S5 shows typical usage of the `read_scan()` function.

Table S2. Attributes and methods from an instance of a `KMCOutput` used to extract simulation data.

Name	Description	Example usage `out = KMCOutput()`	Example output
`area`	Lattice surface area (Å²)	`out.area`	`52.36`
`av_coverage`	Weighted-average coverage of each surface species (%).	`out.av_coverage['CO']`	`12.5`
`av_coverage_per_site_type`	Weighted-average coverage (%) of surface species on each site type.	`out.av_coverage_per_site_type['br']['CO']`	`8.3`
`av_energy`	Weighted-average lattice energy (eV·Å⁻²).	`out.av_energy`	`-0.185`
`av_total_coverage`	Weighted-average total coverage of all surface species (%).	`out.av_total_coverage`	`38.4`
`av_total_coverage_per_site_type`	Weighted-average total coverage (%) on each site type.	`out.av_total_coverage_per_site_type['br']`	`53.2`
`coverage`	Coverage of each surface species over time (%).	`out.coverage['CO']`	`[10.2, 10.3, 10.7, ...]`
`coverage_per_site_type`	Coverage (%) of each surface species on each site type over time.	`out.coverage_per_site_type['bridge']['H']`	`[6.1, 6.1, 6.3, ...]`
`dominant_ads`	Most dominant surface adsorbate across all site types.	`out.dominant_ads`	`"CO"`
`dominant_ads_per_site_type`	Most dominant surface adsorbate for each site type.	`out.dominant_ads_per_site_type['top']`	`"CO"`
`energy`	Lattice energy (eV·Å⁻²) recorded over time.	`out.energy`	`[-0.195, -0.190, -0.187, ...]`
`final_energy`	Lattice energy (eV·Å⁻²) at the final simulation time.	`out.final_energy`	`-0.186`
`finaltime`	Final simulation time (s).	`out.finaltime`	`1.62e4`
`gas_specs_names`	Names of the gas-phase species defined in the simulation.	`out.gas_specs_names`	`["CO", "CO2"]`
`get_selectivity( main_product, side_products)`	Computes selectivity (%) of the *main* product relative to a list of *side* products.	`out.get_selectivity("CH4", ["CO2", "CO"])`	`76.4`
`n_gas_species`	Number of gas-phase species.	`out.n_gas_species`	`2`
`nevents`	Cumulative number of KMC events at each recorded step.	`out.nevents`	`[10000, 20000, 30000, ...]`
`n_sites`	Total number of lattice sites used in the simulation.	`out.n_sites`	`200`
`n_surf_species`	Number of surface-adsorbed species.	`out.n_surf_species`	`3`
`production`	Cumulative production of each gas-phase species over time (number of molecules).	`out.production['CO']`	`[0, 15, 38, 60, ...]`
`site_types`	Mapping of site-type names to the count of each type	`out.site_types`	`{'top': 60, 'bridge': 140}`
`surf_specs_names`	Names of the surface-adsorbed species defined in the simulation.	`out.surf_specs_names`	`["H", "CO", "OH"]`
`time`	Simulation time points (in seconds) at each recorded step.	`out.time`	`[0.0, 3.2, 5.1, 8.4, ...]`
`tof`	Turnover frequency for each gas-phase species (molec.·s⁻¹·Å⁻²).	`out.tof['CO2']`	`1.24e-03`
`total_coverage`	Sum of all surface species coverages (%) over time.	`out.total_coverage`	`[35.6, 37.8, 38.1, ...]`

total_coverage_per_site_type	Sum of coverages (%) of all species on each site type over time.	out.total_coverage_per_site_type['br']	[24.5, 25.0, 24.9, ...]
total_production	Total production of each gas-phase species by the end of the simulation (molecules).	out.total_production['CO']	2345.0

```
from zacrostools.read_scan import read_scan

df = read_scan(
    scan_path='/path/to/scan/folder',
    analysis_range=[50, 100],
    range_type='time',
    weights='time')

df.to_csv('scan_results.csv')
```

Code snippet S5. Reading a collection of simulation outputs, aggregating them into a Pandas DataFrame, and saving the results to a CSV file.

Section S6. Steady state analysis and automatic detection of issues

The `detect_issues` function (introduced in Section 3.2.3 of the main text) helps assess whether a selected `analysis_range` is appropriate for extracting reliable averages. It does so by examining two criteria: the slope of the lattice energy and the linearity of simulated time versus the number of KMC events. At steady state, the lattice energy should fluctuate around a constant value rather than exhibit a sustained upward or downward trend. Similarly, the total rate constant (i.e., the sum of the rate constants for all possible events) should also stabilize, resulting in a linear increase of simulation time with the number of KMC steps. If either of these conditions is not met, the simulation may not have reached steady state. The typical usage of `detect_issues` is illustrated in Code Snippet S6:

```
from zacrostools.detect_issues import detect_issues

detect_issues(job_path='/path/to/simulation_files',
              analysis_range=[50, 100],
              range_type='time')
```

Code snippet S6. Detecting potential issues in a simulation.

If either the energy slope or the linearity criterion is violated, `detect_issues` returns `True`, indicating that the simulation may need to run longer. However, other factors such as catalyst poisoning or instabilities in the stiffness scaling algorithm could also be responsible.

This function accepts the same arguments as `KMCOutput` (`job_path`, `analysis_range`, and `range_type`) and offers two additional optional parameters:

- `energy_slope_thr`: absolute threshold for the energy slope (in eV/Å², default is 5×10^{-10}),
- `time_r2_thr`: r^2 threshold for the linear regression of simulation time vs. number of events (default is 0.95).

Figure S1 presents the lattice energy (top) and simulation time (bottom) as functions of the number of KMC events for two simulations. In Simulation A (left), steady state is reached after ~50% of the total events, while in Simulation B (right), steady state is achieved after only ~10%. Using `detect_issues` with `analysis_range=[20,100]` and `range_type='nevents'` would yield `True` for Simulation A (indicating issues) and False for Simulation B (indicating steady state), assuming the default thresholds.

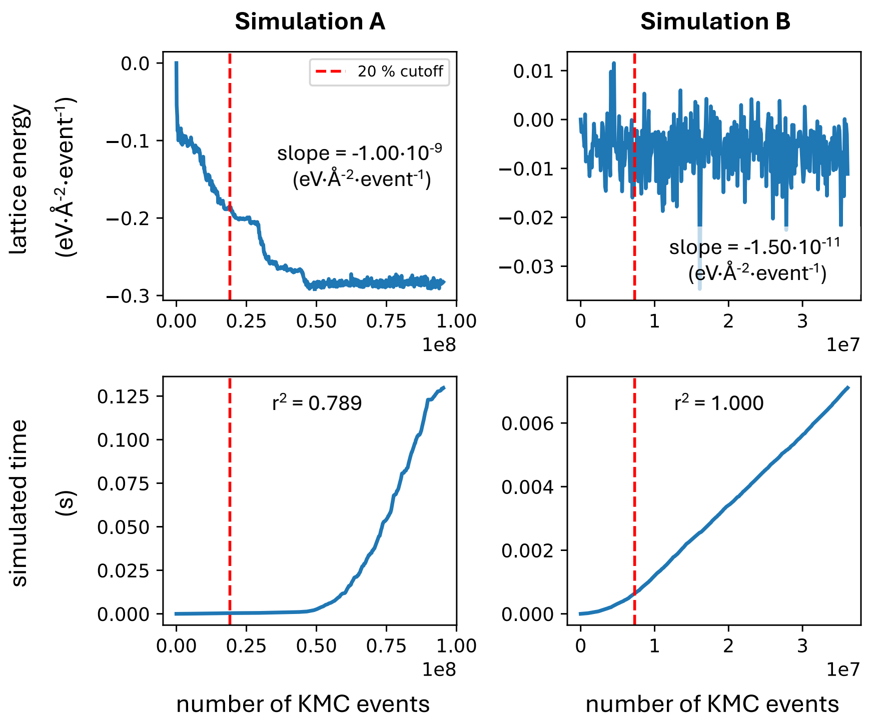

Figure S1. Lattice energy (top) and simulation time (bottom) as a function of the number of KMC events for two different KMC simulations. The plots show the slope of the lattice energy vs. events fit and the r^2 of the time vs. events fit, calculated over the final 80% KMC events (to the right of the dashed red line at 20% of total events).

Section S7. Plotting heatmaps

Code snippet S7 demonstrates how to create various 2D heatmaps from large KMC parameter scans. Common arguments to these plotting functions include `x`, `y`, and `scan_path`, while other parameters (e.g., `main_product` and `side_products`) may be specific to a particular plotting function.

```
import numpy as np
import matplotlib.pyplot as plt
from zacrostools.heatmaps.tof import plot_tof
from zacrostools.heatmaps.coverage import plot_coverage
from zacrostools.heatmaps.phasediagram import plot_phasediagram
from zacrostools.heatmaps.selectivity import plot_selectivity
from zacrostools.heatmaps.finaltime import plot_finaltime
from zacrostools.heatmaps.dtof import plot_dtof

scan_path = '/path/to/main/scan'
scan_path_ref = '/path/to/reference/scan'  # for ΔTOF plot

# Plot parameters
x = 'pressure_CH4'
y = 'pressure_CO2'
analysis_range = [50, 100]
range_type = 'time'
weights = 'time'
min_molec = 10  # for TOF and selectivity plots

tick_labels = {  # for phase diagram plots
    '$CH_{x}$': ['CH3', 'CH3_Pt', 'CH2', 'CH2_Pt', 'CH',
                 'CH_Pt', 'C', 'C_Pt'],
    '$CHO/COH$': ['CHO', 'CHO_Pt', 'COH', 'COH_Pt'],
    '$CO$': ['CO', 'CO_Pt'],
    '$COOH$': ['COOH', 'COOH_Pt'],
    '$CO_{2}$': ['CO2', 'CO2_Pt'],
    '$H$': ['H', 'H_Pt'],
    '$H_{2}O$': ['H2O', 'H2O_Pt'],
    '$OH$': ['OH', 'OH_Pt'],
    '$O$': ['O', 'O_Pt']}

fig, axs = plt.subplots(4, 3, figsize=(9, 8),
                        sharex='col', sharey='row')

for i, product in enumerate(['CO', 'H2', 'H2O']):

    plot_tof(ax=axs[0, i], x=x, y=y, scan_path=scan_path,
             gas_spec=product,
             min_molec=min_molec,
             weights=weights,
             levels=np.logspace(-1, 4, num=11),
             analysis_range=analysis_range,
             range_type=range_type)

for i, site_type in enumerate(['tC', 'tM', 'Pt']):

    plot_coverage(ax=axs[1, i], x=x,y=y, scan_path=scan_path,
                  surf_spec='all',
                  site_type=site_type,
                  weights=weights,
                  analysis_range=analysis_range,
                  range_type=range_type)

    plot_phasediagram(ax=axs[2, i], x=x, y=y, scan_path=scan_path,
        site_type=site_type,
        min_coverage=50.0,
        tick_labels=tick_labels,
        weights=weights,
        analysis_range=analysis_range,
        range_type=range_type)

plot_selectivity(ax=axs[3, 0], x=x, y=y, scan_path=scan_path,
```

```
                   main_product='H2',
                   side_products=['H2O'],
                   min_molec=min_molec,
                   weights=weights,
                   analysis_range=analysis_range,
                   range_type=range_type)

plot_finaltime(ax=axs[3, 1], x=x, y=y, scan_path=scan_path,
               levels=np.logspace(-5, 7, num=13))

plot_dtof(ax=axs[3, 2], x=x, y=y, scan_path=scan_path,
          gas_spec='H2',
          scan_path_ref=scan_path_ref,
          difference_type='absolute',
          scale='log',
          min_molec=min_molec,
          weights=weights,
          analysis_range=analysis_range,
          range_type=range_type)

plt.tight_layout()
plt.show()
```

Code snippet S7. Generating heatmaps for multiple KMC simulations. For complete descriptions of each function's parameters, check the official documentation.[1]

REFERENCES

[1] ZacrosTools documentation; Prats, H. https://zacrostools.readthedocs.io/en/latest/ (accessed May 25, 2025).
[2] Sprowl, L. H.; Campbell, C. T.; Árnadóttir, L. Hindered translator and hindered rotor models for adsorbates: Partition functions and entropies. *J. Phys. Chem. C* **2016**, *120*, 9719-9731.
[3] Stamatakis, M.; Vlachos, D. G. A graph-theoretical Kinetic Monte Carlo framework for on-lattice chemical kinetics. *J. Chem. Phys.* **2011**, *134*, 214115.
